# Polyimide Layers with High Refractivity and Surface Wettability Adapted for Lowering Optical Losses in Solar Cells

**DOI:** 10.3390/polym14194049

**Published:** 2022-09-27

**Authors:** Andreea Irina Barzic, Raluca Marinica Albu, Camelia Hulubei, Samy F. Mahmoud, Ola A. Abu Ali, Zeinhom M. El-Bahy, Iuliana Stoica

**Affiliations:** 1 Department of Physical Chemistry of Polymers, “Petru Poni” Institute of Macromolecular Chemistry, 41A Grigore Ghica Voda Alley, 700487 Iasi, Romania; 2 Department of Biotechnology, College of Science, Taif University, P.O. Box 11099, Taif 21944, Saudi Arabia; 3 Department of Chemistry, College of Science, Taif University, P.O. Box 11099, Taif 21944, Saudi Arabia; 4 Department of Chemistry, Faculty of Science, Al-Azhar University, Nasr City 11884, Cairo, Egypt

**Keywords:** polyimide, optical properties, morphology, wettability, solar cells

## Abstract

The performance of photovoltaics with superstrate configuration is limited by the rigidity and low refractivity of a classical glass cover. In this work, two polyimides (PIs) and two copolyimides combined in the main chain cycloaliphatic moieties, aromatic sequences, chalcogen atoms, and having/lacking fluorine atoms, are proposed as shielding covers for solar cells. The samples containing small cycloaliphatic moieties displayed high transmittance above 80% at 550 nm. The refractive index values under changeable wavelengths and temperatures were shown to influence the magnitude of the reflection losses. At the sample interface with the transparent electrode, optical losses were reduced (~0.26%) in comparison to the classical glass (~0.97%). The samples with the best optical features were further subjected to a surface treatment to render the self-cleaning ability. For this, a new approach was used residing in irradiation with the diffuse coplanar surface barrier discharge (DCSBD), followed by spraying with a commercial substance. Scanning electron microscopy and atomic force microscopy scans show that the surface characteristics were changed after surface treatment, as indicated by the variations in root mean square roughness, surface area ratio, and surface bearing index values. The proposed PI covers diminish the optical losses caused by total internal reflection and soiling, owing to their adapted refractivity and superhydrophobic surfaces (contact angles > 150°), and open up new perspectives for modern photovoltaic technologies.

## 1. Introduction

Solar radiation is not only the supporting key factor of life on our planet but it can also be regarded as an excellent source of clean energy production [1]. The motivation behind the utilization of Sun-emitted energy is linked to the goal of the diminishment of pollution caused by classical energy from degradable fuels. In this context, photovoltaic devices have an outstanding technical evolution and current demands are focused on cost reduction, increased conversion efficiency, eco-friendly nature, lightweight properties, and flexibility [2,3]. The latter two features are limited in the case of thin film solar cells by the presence of the external cover glass, which is very rigid and weighs around 70% of the whole device [4]. From this point of view, polymers seem to be the key solution given their excellent flexibility and ease of processing into thin films or layers [4,5]. In addition to this, all device components are constantly subjected to solar radiation heating; thus, thermal stability is an essential element that impacts power conversion efficiency [6]. Other problems that reduce the efficiency of a solar cell through optical losses are determined by two important aspects. The first one is represented by the inconsistency among the refraction features of the glass in regard to those of the neighboring media, which narrow the percent of the entering radiation at the air/glass interface (4%) and glass/electrode (1.38%) interface by total internal reflection (TIR). As a consequence, a part of Sun-emitted radiation does not pass beyond the glass layer and is turned back because of the distinct values of the refractive index [7]. The same phenomenon occurs for the radiation arriving at the glass/transparent electrode interface. Hence, the quantity of reflected radiation is enhanced for steeper angles and, thus, less light interacts with the junction of the photovoltaic cell, diminishing its power output. The second aspect resides in the soiling of the sunlight receiving surface of the solar cover glass [8], which impedes the incident radiation to enter inside the layered structure of the solar cells, generating a lower efficiency by such optical losses. Mainly, dust accumulation is the reason for scanty output power and is rinsed out in the rainy season, leading to an energy gain ranging between 3.0 and 4.3% during a year [9,10]. The deposition of dirt particles on the solar module favors reactions between them and the glass cover, which complicates the cleaning process. This is another reason that requires the replacement of the glass cover with new anti-soiling coatings. Nayshevsky et al. [8] revealed that, by means of the polymer wetting features, it is possible to control the soiling rate and the cleaning efficiency under the influence of condensed water. According to their investigation [8], the development of highly hydrophobic polymer surfaces could resolve the problem caused by optical losses produced by soiling. 

All of the aforementioned aspects must be accounted for when selecting a material for photovoltaic shielding. One category of polymers that best meets the described technical demands is represented by polyimides (PIs), which are acknowledged for their capacity to remain stable under heat, moisture, chemical substances, and radiation. The balance between the PI’s thermal, optical, morphological, and wettability characteristics can be adjusted by means of the chemical structure [11,12]. The literature shows that there are not many studies dealing with PI involvement in solar cell applications and they principally describe fully aromatic commercial structures (Kapton [13] or Upilex [14]), while others analyze certain aromatic non-commercial PIs [15,16], all being employed as flexible substrates. The first study dealing with the utility of aromatic PIs as flexible shielding layers for solar cells was conducted by Feenstra et al. [4]. However, their report focused on transparency in the presence of Ce dopants and failed to discuss the optical losses caused by refraction mismatch or other factors. The idea of using polyimides containing alicyclic units as covers for solar cells was recently proposed by our research group [17]. As far as we know, in the literature, no study before ours discussed the reduction of optical losses by means of refractive index adjustment of the PI solar cell cover. The major issue of aromatic PIs comes from the limited transparencies at optical wavelengths [18] that restrict their utilization in photovoltaic devices. Related to this, it has been proven that incorporating fluorine groups [19] or alicyclic segments [20] of the PI backbone enhances transparency, while thermal resistance is almost undiminished. However, incorporating low polarizable units reduces the refractive index closer to that of a classical cover glass, which enlarges the TIR-derived optical losses at the cover/electrode interface. The use of highly refractive PIs can be extended to LED devices, which currently are aimed at better performances by incorporating an efficient electron transport layer [21]. PIs can be used as flexible substrates for LEDs due to their thermal resistance and flexibility [22,23]. The lack of polymer layer rigidity is useful for making a new generation of LEDs of practical use in display and lighting commercialization. Luo et al. [24] detailed the effect of flexible substrates, electrodes, and LED architectures on the resulting performance. In their review, they explained the role of flexible polymers in this domain, showing that by using materials that could suffer outstanding mechanical deformation, the applicability of LEDs could be enhanced in regard to the same device placed on the rigid glass supports. Moreover, the optical properties of PIs could be exploited in manufacturing encapsulation materials for LEDs, with a good balance between transparency and refractivity. The latter is needed for expanding the light escape cone, thus upgrading the light extraction efficiency [25].

For both types of applications, it is important to mention that it is hard to obtain PIs with an adequate balance between high transparency and high refractive index. For this goal, in our previous works [26,27,28], a new PI synthesis strategy was proposed, involving the combination of aromatic sequences with aliphatic ones and chalcogen atoms in the main chain. By this procedure, PI materials with a good balance between thermal stability, optical transparency, and refractivity were attained. The chemical structures of the PIs, here under analysis, were previously analyzed by infrared spectroscopy and an energy-dispersive X-ray [27]. Rheology tests of the corresponding solutions revealed a prevalent Newtonian behavior and elastic character at higher shear rates and frequencies, respectively [26,27]. The refractive index was theoretically estimated to range between 1.66 and 1.69 at 589 nm. Differential scanning calorimetry performed on these PI structures was as in [27], indicating that the vitrification temperature (T_g_) ranged between 204 and 342 °C as a function of the chemical structure, particularly the degree of chain rigidity and intermolecular interactions. Thus, the biggest T_g_ values were recorded for the polymers containing rigid CBDA units, which are able to constrain the chain segment relaxation and movements. Thermogravimetry experiments revealed that these polymers displayed high initial degradation temperatures (>420 °C), which showed remarkable thermal stability. This property is known to be advantageous for solar cell applications. 

This article continues previous efforts by providing a deeper exploration of the optical properties on a wider wavelength and temperature interval. The experiments were meant to provide original insights into the relationship between the PI’s chemical structure/dispersion properties in regard to the minimization of optical losses at the interface of the PI layer with both air and conductive electrodes. It will be demonstrated that the dependence of the refraction index on the wavelength and temperature and the matching of the dispersion curves with those of adjacent media are essential aspects for proper light management in solar cells. Aside from TIR-related optical losses, this work is also concerned with solving the issue of losses produced via light absorption by impurities deposited in time on top of the device. For this, a new facile strategy is proposed to adapt to the surface of the PI layer (in contact with air) that consists of a surface activation by plasma, followed by the linking of a highly hydrophobic substance, which renders self-cleaning features to the PI cover. Morphological characteristics at the nanometric level were studied by atomic force microscopy and their evolution with the surface adaptation steps was monitored. Illuminance measurements confirmed that light passed properly through the modified PI films. Dimensional stability was evaluated by calculating the coefficient of thermal expansion (CTE). The major contribution of this paper is that it proposes new PI shielding layers that, for the first time, concomitantly address the optical losses arising from TIR and soiling by means of their refraction, morphological, and wettability properties. In this way, a larger percentage of incident radiation will reach the active area of the solar cell, making improvements to the conversion efficiency. 

## 2. Materials and Methods

### 2.1. Materials

**Figure 1:**
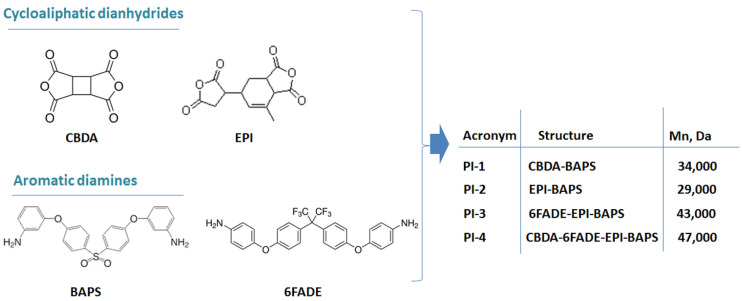
Schematic illustration of the monomer structures. The inserted table contains information on the acronyms, the polyimide structures after the monomer combination, and the number of average molecular weights corresponding to each sample.

The synthesis procedure of the polyimides from this study was published in a previous work [27]. Briefly, a two-stage polycondensation approach was employed to develop the new polyimide structures. Initially, the monomers were mixed in N-methyl-pyrrolidone (NMP) under ambient temperature and a 15 wt% solid content was kept. Secondly, the system was subjected to specific heating conditions (185 °C) to produce the thermal imidization reaction under nitrogen flow, which rendered the designed polyimide structure. The first sample, denoted PI-1, was obtained based on the reaction of 1,2,3,4-cyclobutanetetracarboxylic dianhydride (CBDA) with bis [4-(4-aminophenoxy) phenyl] sulfone (BAPS). The second sample, denoted PI-2, was the result of the same diamine BAPS that reacted with 5-(2,5-dioxotetrahydrofuryl)-3-methyl-3-cyclohexene-1,2-dicarboxylic anhydride (EPI). The third sample denoted, PI-3, was attained based on EPI dianhydride in combination with two diamines, namely BAPS and 2,2-bis [4-(4-aminophenoxy)phenyl]hexafluoropropane (6FADE). The fourth sample, denoted PI-4, was attained by using a pair of two dianhydrides, CBDA and EPI, reacting with two diamines, BAPS and 6FADE. Figure 1 displays a clear correspondence between the sample structure and ascribed acronym. 

The PI films were achieved as previously reported [27] by the deposition of the PI precursor solution on clean supports, followed by a specific thermal treatment. Upon finalizing the imidization step, the PI films were removed from the supports by soaking in water and then drying. Then, the samples were subjected to surface adaptation by following a new two-stage approach: (1) the PI film was exposed to diffuse coplanar surface barrier discharge (DCSBD) plasma for 2 min using an RPS40 system; (2) the plasma-activated PI surface was sprayed with the Gardx Protection Stain Guard commercial product to induce a pronounced hydrophobic character to the samples. 

The average molecular weight (Mn) of the samples was measured by gel permeation chromatography and the values were comprised between 29,000 and 47,000 Da (see Figure 1) with a PDI smaller than 1.7.

### 2.2. Methods

Transparency of the polyimide samples was tested on a SPECORD 210 PLUS device (Analytik Jena GmbH, Jena, Germany).

Refractometry data were collected on the Abbemat MW device (Anton Paar GmbH, Ashland, VA, USA) at variable temperatures (25–65 °C) and wavelengths (486–670 nm). The accuracy of the refractive index was 10^−5^.

The illuminance properties of the pristine and surface-modified PI samples were determined on a CL-70F (Konica Minolta, INC., Tokyo, Japan) device.

Wettability was tested by measuring the contact angles made by water drops on the pristine and modified surface of the PI films. Five repetitive measurements were made on each sample, the error being around ±1°.

Atomic force microscopy (AFM) measurements were made in tapping mode using a NTEGRA scanning force microscope with Nova 19891 software purchased from NTMDT Spectrum Instruments (Zelenograd, Moscow, Russia). The cantilever type was NSG10 (NTMDT Spectrum Instruments, Zelenograd, Moscow, Russia). Its measured resonant frequency was 162 kHz. Employing the algorithms of normal and torsional spring constant determination developed by John E. Sader [29], the values of 7.3 N/m and 9.7 × 10^−10^ N/m were obtained for the normal and torsional spring constants, respectively. The scanning areas were 65 × 65 µm^2^ and 20 × 20 µm^2^, these being chosen to better highlight the morphological characteristics on the surface. Image Analysis 3.5.0.19892 software was used to compute the root mean square roughness (Sq), surface area ratio (Sdr), and surface bearing index (Sbi) [30]. The average dimensions of the pores and the corresponding standard deviations were calculated using a pore analysis.

Scanning electron microscopy (SEM) tests were made on a Verios G4 UC device (Thermo Scientific, Prague, Czech Republic) equipped with a concentric (insertable) backscatter electron detector (CBS) at an accelerating voltage of 10 kV, performed in high vacuum mode. The samples were coated with 10 nm of platinum using a Leica EM ACE200 (Leica Microsystems, Wetzlar, Germany) sputter-coater to provide electrical conductivity and prevent a charge buildup during exposure to the electron beam. 

## 3. Results and Discussion

The performance of a photovoltaic cell can be further improved by using this newly proposed strategy that aims to control both refraction and surface properties for the diminishment of the optical losses produced by TIR and soiling issues. For better light capturing inside the device, it is essential to understand PI refractivity variation with the wavelength and temperature and to enhance surface hydrophobicity to avoid light absorption by the impurities on the device surface. In this way, a larger amount of incident radiation will pass to the active zone and increase the conversion efficiency.

### 3.1. Optical Analysis and Related Losses

Optical properties, including transmittance and refractive indices, are key factors in determining the fitting of a polymer candidate for a solar cell cover. Absorption or TIR-determined losses are undesirable for such an application. This study aims first to perform experiments regarding these optical features and, secondly, based on the optical performance, to discern the level of suitability of the synthesized PI structures for the pursued scope.

#### 3.1.1. Transparency and Absorption Edges

**Figure 2:**
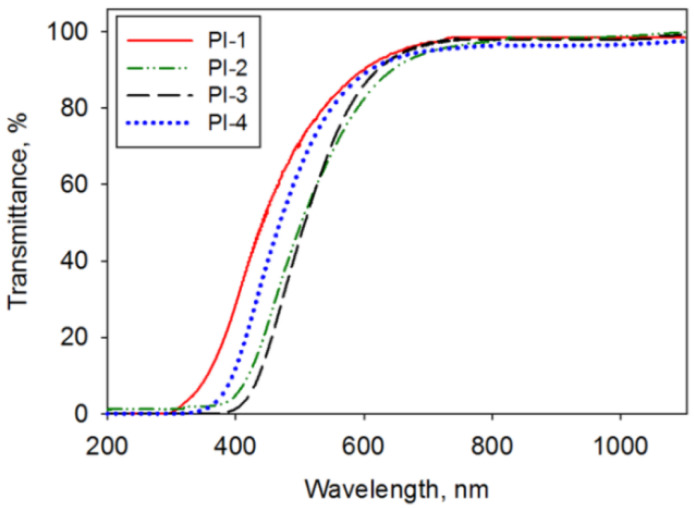
The UV-VIS-NIR spectra recorded for the PI-1, PI-2, PI-3, and PI-4 films.

For a polymer solar cell cover, it is mandatory to have a large fraction of incident light that travels through the material and reaches the other side towards the transparent electrode. The transmittance data for all studied PIs are recorded in the UV-VIS-NIR domain, as depicted in Figure 2. 

The prepared polymers exhibited good transparency in the visible range, namely at 550 nm, the transmittance was 82.71% for PI-1, 68.58% for PI-2, 70.71% for PI-3, and 80.26% for PI-4. The results are similar to the transparency reported for other PIs containing BAPS moieties [31,32]. Among the analyzed PI films, it can be noted that a higher amount of incident radiation comes out of the other side of PI-1 and PI-4 samples, particularly under 600 nm. So, from this point of view, these two samples better fulfilled the optical transparency requirement imposed for the solar cell cover application.

The absorption coefficient (α) could be further determined by introducing the transmission data in Formula (1):

α = (1/t) ∙ ln(1/T) (1)

where T stands for the transmittance and t is the thickness of the investigated film.

**Figure 3:**
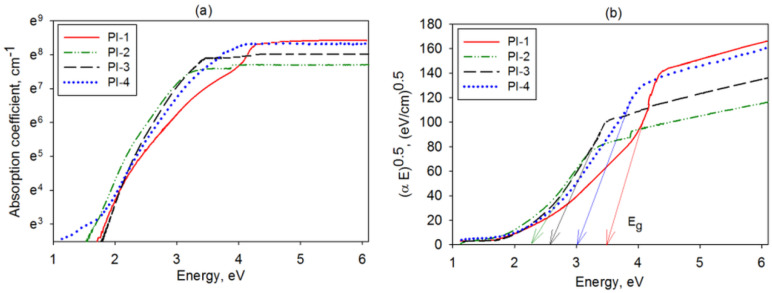
(**a**) The absorption coefficient variation with energy and (**b**) Tauc plot for the PI-1, PI-2, PI-3, and PI-4 films.

The attained absorption coefficient variation with the photon energy (E) is illustrated in Figure 3a for all samples. The shapes of the plots resemble those suggested by Tauc and Menth [33], while the slopes of the exponential edges follow Urbach, Formula (2) [34]:

(2) $$\ln\;\alpha ={\ln\alpha }_{0}+E/E_{u}$$

where α_0_ is a constant, while E_u_ is the Urbach energy.

The semi-logarithmic dependence of α on E, shown in Figure 3a, allowed the evaluation of the Eu parameter, which was found to be 460.8 meV for PI-1, 401.6 meV for PI-2, 377.4 meV for PI-3, and 425.5 meV for PI-4. Based on these data, it can be stated that larger disorders were obtained for PI-1 and PI-4 samples due to potential structural defects (e.g., break, torsion of the macromolecular chains), which are known to be responsible for the Urbach-like feature of the absorption edge. The E_u_ results are in agreement with those obtained for other PIs containing aliphatic segments [35].

Tauc’s approach [33] reveals that the optical band gap energy can be acquired from the examination of the linear part of the plot described by Formula (3):

(3) $$\alpha E=D{\left(E-E_{g}\right)}^{m}$$

where D stands for a constant, E_g_ means the optical band gap, and m denotes an index whose value is typical for certain transitions. 

For polymers, an adequate fit was attained for m = 1/2 (allowed direct transitions), rendering a linear dependence, which was recognized as being the Tauc relation [33]. For the studied PI films, the Tauc plots are shown in Figure 3b. When performing the extrapolation of the regression line accomplished by the least squares estimation method to (αE)^1/2^ = 0 coordinate, it was possible to estimate the optical band gap energy. 

For the PIs under analysis, E_g_ ranged as follows: 3.46 eV (PI-1) > 3.03 eV (PI-4) > 2.58 eV (PI-3) > 2.27 eV (PI-2). The differences in the magnitude of the optical band gap indicate that the sharp discontinuity in the absorption spectrum appeared at distinct wavelengths; the energy ascribed to the absorbed light quantum was attributable to an electronic transition. Therefore, the PI-2 and PI-3 samples displayed absorption discontinuities, which were noticeable at higher wavelengths, while for PI-1 and PI-4, they were remarkable at smaller ones. Moreover, the higher threshold for photon absorption for PI-1 and PI-4 supports the reduced probability of electronic transitions and the good transparency of these polymers. This is additionally sustained by the work of Jarzabek et al. [35], who revealed that the materials having an E_g_ beyond 3 eV could be considered transparent, being suitable for the pursued scope.

#### 3.1.2. Refraction and TIR-Induced Losses

**Figure 4:**
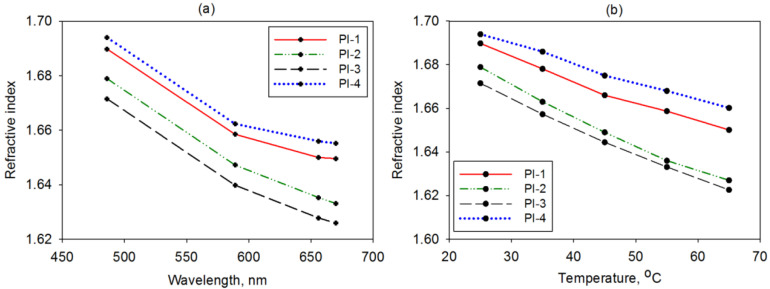
The variation of the refractive index with (**a**) a wavelength at 25 °C and (**b**) temperature at 486 nm for the PI-1, PI-2, PI-3, and PI-4 films.

The refractive characteristics at variable wavelengths and temperatures are paramount for quantifying the amount of solar radiation penetration through the solar cell layers. For such a purpose, the refractive index (n) of the studied PI covers was experimentally measured; the results are delivered in Figure 4a,b. The examined polymer foils displayed a decreasing refractive index as the radiation wavelength increased, as shown in Figure 4a. At 25 °C, the magnitude of this optical parameter (for all wavelengths) ranged as follows: PI-3 < PI-2 < PI-1 < PI-4. This could be attributable to the differences in the polarizability of the PI structure. For instance, the incorporation in the main chain of the small CBDA moieties (samples PI-1 and PI-4) led to a higher density of polar imide rings and, hence, larger polarizability and a higher refractive index. The values of the refractive index of the examined PIs were higher than that of other PIs containing chalcogen atoms, which had n~1.617 [36]. On the other hand, the refractive features of the PI covers were influenced by environmental temperatures at which the solar cell operated. Figure 4b shows the refractive index change of the PI-1–PI-4 samples with this factor at 486 nm. For all samples, the values of the refractive index were diminished upon increasing the temperature from 25 °C to 65 °C. The rate of variation of n with temperature was smaller for the studied PIs compared to that of the PIs with chalcogen atoms reported by Rosenberg [36]. 

**Figure 5:**
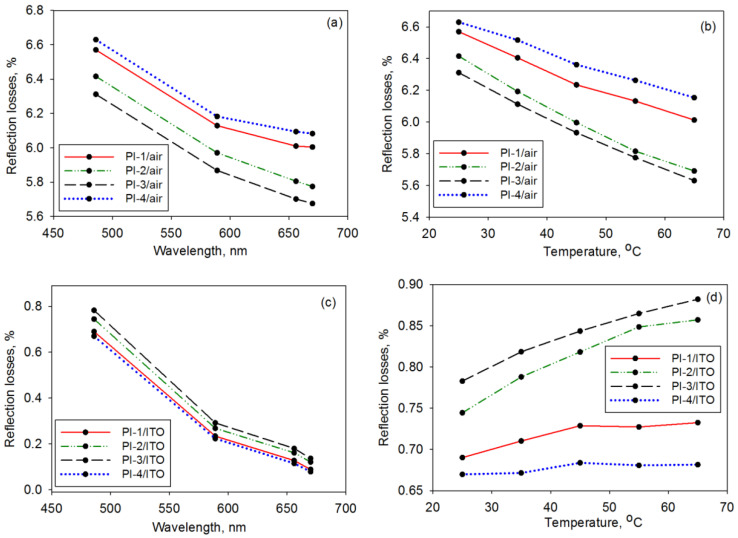
The wavelength and temperature dependence of the reflection losses for the PI-1, PI-2, PI-3, and PI-4 films at (**a**,**b**) the air interface and (**c**,**d**) ITO interface.

To increase the efficiency of a solar cell, it is important to enhance the quantity of penetrating solar radiation into the active zone. Since the materials composing the photovoltaic device have distinct optical features, light passing through each interface might be reflected into the incident medium if the refractive index difference is too large. Therefore, to minimize the reflection losses (R%), it is mandatory to avoid TIR. The latter aspect is determined by the refractive characteristics of the upper layers of the device, namely the PI cover and the transparent electrode often made of indium tin oxide (ITO) [37]. The problem arises from the fact that the classical cover glass has a refractive index that is too small in regard to that of the ITO electrode [38]. Based on the recorded optical features, the reflection losses at normal incidences were evaluated at the PI cover interface with both air and ITO, as shown in Figure 5. 

The obtained data reveal that the PI structure determines the variations in the reflection losses, and the temperature and wavelength produce significant changes at R%. At the air/PI interface, it can be seen in Figure 5a,b that the reflection losses were around 6.6% since the polymers had a more discrepant refractive index in comparison to that of air. The enhancements of both wavelength and temperature determined the reduction of the R% magnitude at 0.69% and 0.48%, respectively. At the PI/ITO interface (Figure 5c,d), the optical losses were reduced (around 0.26% at 589 nm) in regard to those accumulated at the interface of ITO with a classical glass cover (~0.97%). Since the refraction features of ITO were better matched with PI-1 and PI-4, the reflection losses were smaller. The PI structure determined a more pronounced difference in R% values when the temperature varied. The slight increase of the reflection losses for all samples under changing temperatures could be ascribed to the fact that the refractive index of ITO had a less abrupt variation with the temperature than the PI samples. At the sample/ITO interface, the R% values range was ~0.64% for the variable wavelength and ~0.02% for variable temperatures. In summary, the latter factor determined a smaller impact on R% than the light wavelength. The refractive features of PI-1 and PI-4 allowed a larger percentage of radiation to pass toward the active layer of the photovoltaic cell, which was beneficial for improving the conversion efficiency.

### 3.2. Evaluation of Coefficient of Thermal Expansion

The coefficient of thermal expansion (CTE) is an important factor that provides information on the dimensional stability of the studied polymers. In electronics and many technical applications, it is important to know the extent to which the unrestrained polymer layer that is heated to a certain temperature is able to keep its initial dimensions. The procedure elaborated by Bicerano [39] allows estimating the CTE of a polymer starting from its structural characteristics, as shown in Equation (4):

(4) $$\mathrm{CTE}=\frac{1}{289+9.47\cdot T_{g}}$$

where CTE is the coefficient of thermal expansion and T_g_ is the vitrification temperature.

Based on previous results regarding the T_g_ magnitude of each PI sample, it is possible to assess CTE. The obtained data are illustrated in Table 1. 

The chemical structure of the studied PIs affected the magnitude of CTE. The largest T_g_ was observed for PI-1 and PI-4 structures, which presented higher rigidities caused by the CBDA presence in the backbone. The obtained CTE values for these polymers were of the same levels of magnitude as those reported for other PIs containing chalcogen atoms [40]. The stronger intermolecular interactions, noted for PI-1 and PI-4, led to smaller values of CTE compared to the other two samples. The lower estimated CTE values were indicative of better stability of CBDA-containing PIs, which is adequate for applications. 

### 3.3. Surface Treatment of the PI Covers

The optical analysis of the samples enabled making adequate selections among the prepared PIs to fulfill the requirements for photovoltaic cover uses. Since the PI-1 and PI-4 samples displayed higher transparency and lower reflection losses, these materials were further subjected to a surface treatment to gain a self-cleaning ability on the side exposed to solar radiation. The employed approach aims to increase the hydrophobic character by means of a new two-step procedure that (1) involves PI exposure to DCSBD plasma, and (2) the activated polymer surface is sprayed with a commercial product containing hydrocarbons, n-alkanes, and iso-alkanes. In the following subsections of the paper, the effects of the surface treatment on the illuminance, morphology, and wettability are shown.

#### 3.3.1. Influence on the Illuminance 

**Figure 6:**
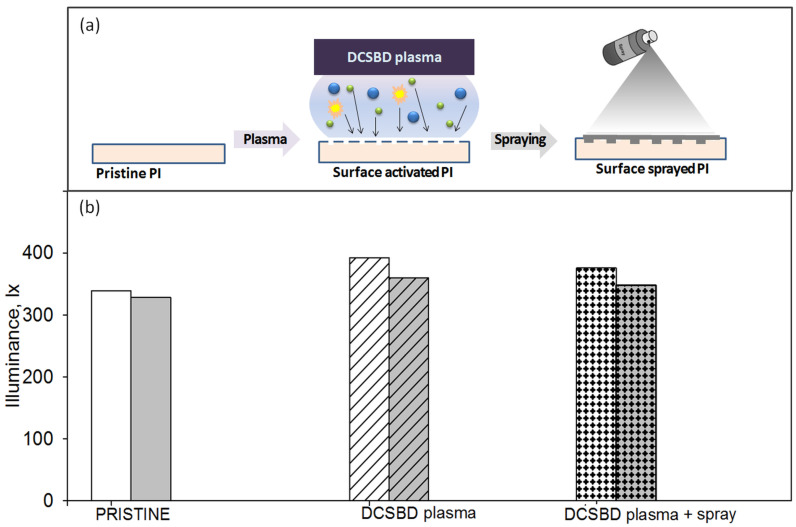
(**a**) The schematic representation of the polymer surface modification approach and (**b**) its influence on the illuminance for the PI-1 and PI-4 samples.

The illuminance represents the overall luminous flux arriving on a material surface, per unit area. In order to verify if the proposed method of the surface adaptation negatively changed the capacity of the PI cover to transmit solar radiation inside the solar cell, the illuminance was experimentally determined. For a clearer understanding of the elaborated methodology for PI hydrophobization, in Figure 6a, the stages of the PI surface modification are depicted. Figure 6b illustrates the changes in illuminance with each step of surface treatment of the PI-1 and PI-4 samples. 

Initially, the illuminance of the PI-1 was slightly higher than that of PI-4, as supported by UV-VIS spectral results. Upon DCSDB plasma irradiation, the illuminance of both samples was enhanced, keeping the same difference between the two PI structures. After the spraying step, the illuminance was reduced but not under the level of the values corresponding to the pristine samples. These measurements confirm that the elaborated surface adaptation protocol does not reduce the extent of how much the incident optical radiation illuminates the PI surface. This is beneficial for the pursued applicative scope. 

#### 3.3.2. Influence of the Surface Treatment on the Morphology 

**Figure 7:**
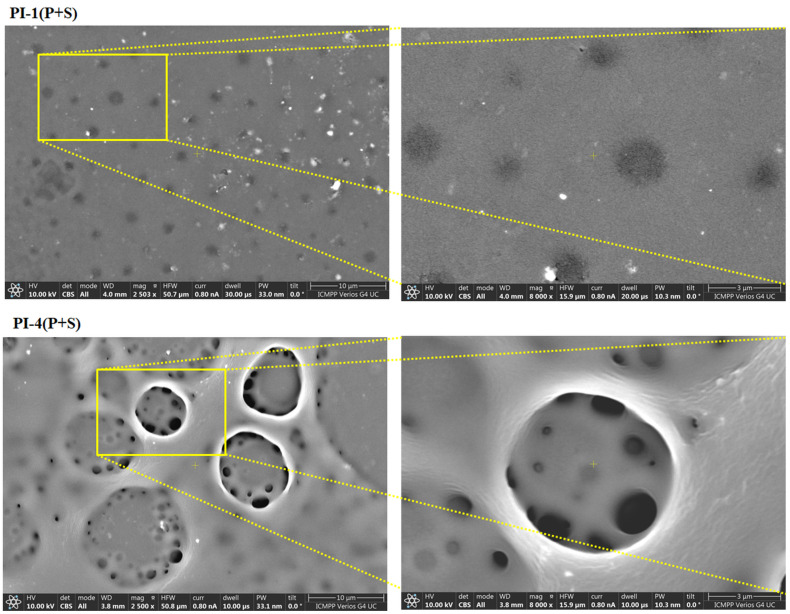
The SEM images of the plasma-treated and -sprayed PI-1(P+S) and PI-4(P+S) films.

The influence of the surface treatment on the surface morphology was investigated by means of SEM and AFM measurements. The morphology of the pristine PI layers was studied at the macro-level by SEM in a previous work [27], where it was remarked that all prepared foils displayed relatively flat surfaces and negligible tiny defects. After plasma irradiation followed by spraying, the PI film surface topography changed, as noted in Figure 7. According to SEM micrographs, both modified PI samples appeared to be covered with a porous layer after depositing on their activated surfaces the hydrophobic compound. It was observable that the spreading ability of the casted substance was dependent on the structure of the PI substrate. Better uniformity of the hydrophobic layer was obtained for the PI-1(S+P) sample. In this case, the pores had smaller dimensions and displayed similar diameters compared to the PI-4(S+P) sample for which the pores had irregularly uneven size features. The morphological aspects were further investigated at the nano-scale by means of AFM experiments.

**Figure 8:**
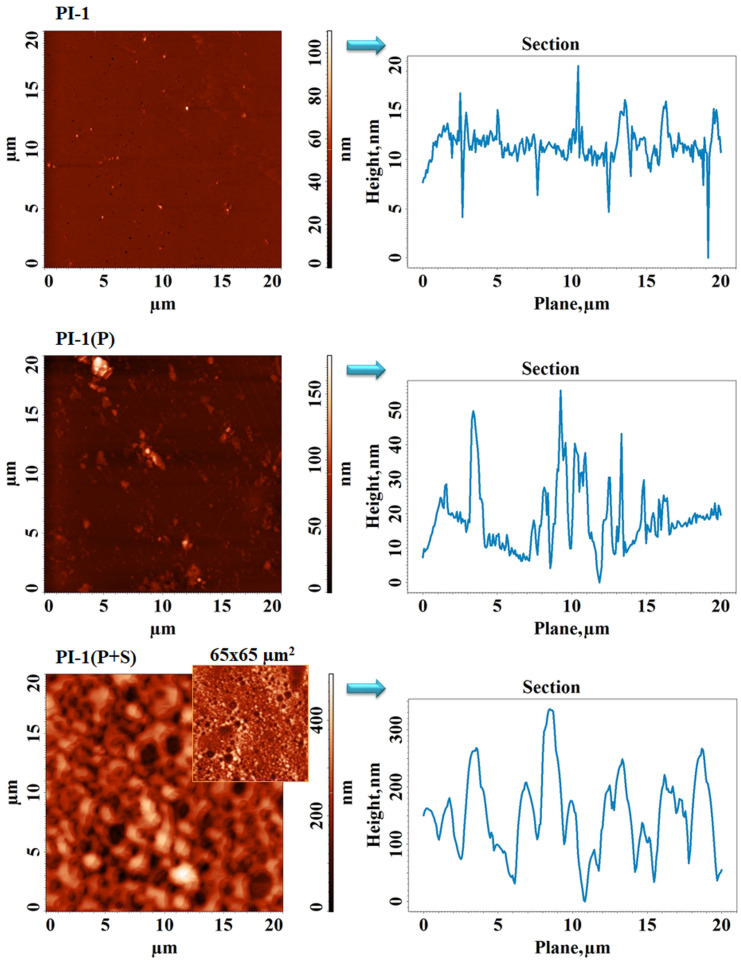
The AFM images and corresponding average cross-section profiles collected for the sample PI-1 before and after the DCSBD plasma treatment (P) and DCSBD plasma treatment followed by spraying (P+S).

**Figure 9:**
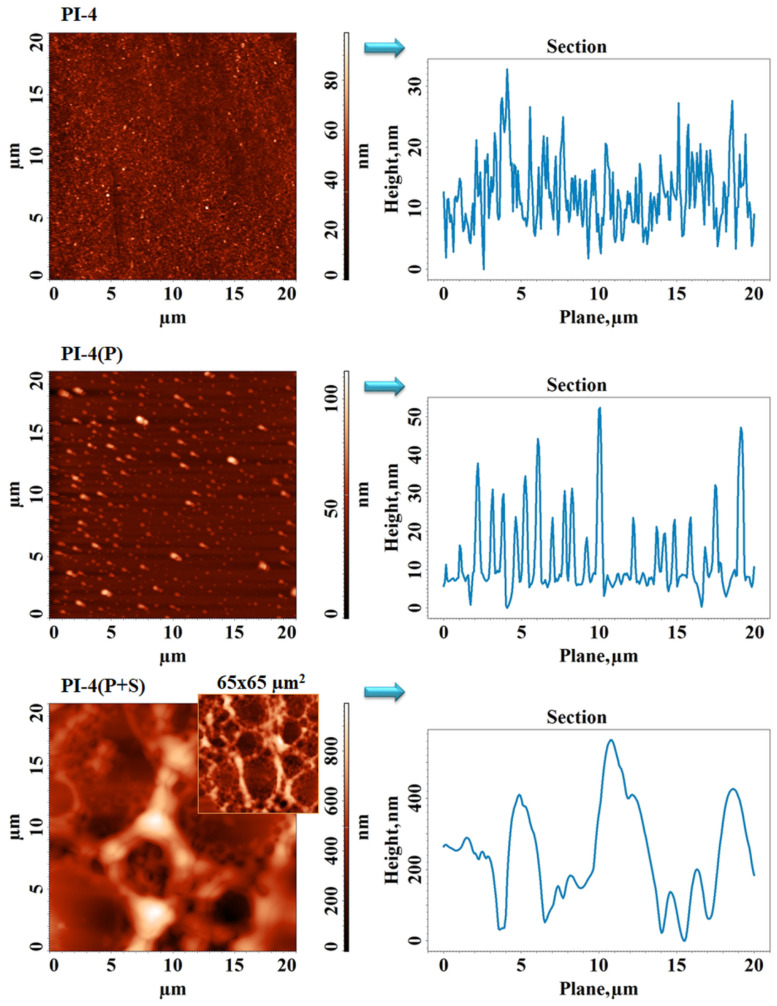
The AFM images and corresponding average cross-section profiles collected for the sample PI-4 before and after DCSBD plasma treatment (P) and DCSBD plasma treatment followed by spraying (P+S).

The AFM images and corresponding average cross-section profiles collected for the investigated PI-1 and PI-4 samples before and after DCSBD plasma treatment (P) and DCSBD plasma treatment, followed by spraying (P+S), are presented in Figure 8 and Figure 9, respectively. The starting samples had surfaces with low values of roughness, 2–5 nm, as seen in Table 2. According to the height distribution on the Z axis placed on the right side of the AFM image, PI-1 showed relief with predominantly low formations, while the relief of PI-4 was mostly made up of globular formations densely and uniformly arranged on the entire surface. For this reason, the complexity of the surface morphology, expressed by calculating the surface area ratio parameter (Table 2), was higher for the copolymer. For the unmodified PI films, it can be noted that the root mean square roughness (Sq) was similar to other PI structures containing alicyclic units and chalcogen atoms in their backbone [41]. After the DCSBD plasma treatment, the resulting chemical species acted differently on the surface, depending on the imide polymer structure. An obvious roughening was observed, especially for PI-1(P) (see Figure 8), in which case, Sq and Sdr increased (even at four–four and a half time) compared to the Sq and Sdr of the control sample. For PI-4(P), the section analysis indicates an increase in size and a diminution in the density of the globular formations, leading to higher roughness, but a lower surface microtexture complexity. Thus, the surfaces of the PI-1(P) and PI-4(P) samples were activated in a different manner. The discussed values for Sq, Sdr, and Sbi surface texture parameters for the pristine- and plasma-irradiated PI films were comparable with the values obtained for other PI structures with chalcogen atoms with/without alicyclic units [42,43]. The general tendency was that the surfaces of the pristine PI films were flat and uniform with a roughness not exceeding 5 nm. Hence, the developed area depicted by the Sdr parameter and the bearing characteristics of the Sbi parameter had small values. Upon the plasma treatments of other PI structures, an increase of these parameters was noted similar to the PIs here under analysis [43]. This was attributed to the fact that plasma etching contributed to a higher developed relief, which was reflected in the bigger values of Sdr and Sbi. The spraying process left a layer on the surface, which was organized as a network with pores. This network was denser/tighter in the case of polyimide PI-1(P+S), the pores being uniform and of 2.2 ± 0.8 µm (medium size) (Figure 8). On the other hand, on copolyimide PI-4(P+S), the mesh was much wider (Figure 9), with pores with larger apertures (12.7 ± 3.4 µm) inside, which organized some other pores with perfectly circular shapes of much smaller sizes (860 ± 203 nm). The bearing area ratio (Table 2) was calculated from the plotted bearing area curve to evaluate the bearing properties of the samples. Initially, Sbi was very small, having values below 0.1, and slightly increased after treatment in the DCSBD plasma. Overall, the final spray-treated samples still had low surface bearing properties since Sbi < 0.608, which is favorable for the pursued application.

#### 3.3.3. Influence of the Surface Treatment on the Wettability

**Figure 10:**
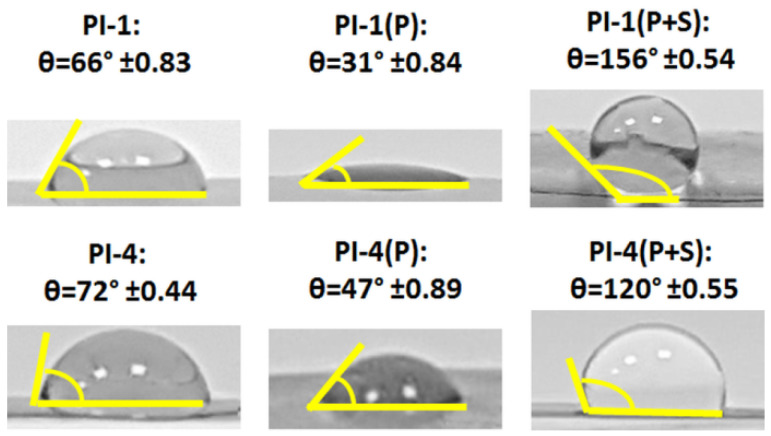
The variations of the contact angle of the pristine PI-1 and PI-4 films, after plasma modification of PI-1(P) and PI-4(P), and after plasma exposure succeeded by spraying PI-1(P+S) and PI-4(P+S).

The surface treatments of the PI-1 and PI-4 were expected to change their wettability behaviors. As remarked in Figure 10, the pristine polymer surfaces were relatively hydrophobic. The water contact angle (θ) of PI-4 was slightly larger than that of PI-1 probably due to the presence of fluorine in its chemical structure. The values of the water contact angle on the pristine PI films were smaller in comparison with those reported for other PIs containing alicyclic units and chalcogen atoms [41]. For both polymers, DCSDB plasma irradiation rendered a certain degree of hydrophilicity (lower water contact angle) as a consequence of the PI surface activation. The latter aspect was favorable for the surface binding of the desired substances. Upon the spraying stage, the contact angle considerably increased beyond 100° for both PIs. In the case of PI-1, the hydrophobic character was more pronounced (θ > 150°) in comparison to PI-4 (θ < 150°). This could be attributed to the fact that plasma could not activate to the same extent as the copolyimide structure, as noted for the homopolyimide, and this was reflected in the distinct interaction with the sprayed hydrocarbon-containing substance. Moreover, the morphological features of both PIs influenced this result. The superhydrophobic surface of the plasma-irradiated and -sprayed PI-1 was suitable for rendering self-cleaning properties, as revealed by the literature data [44]. Moreover, the level of hydrophobicity expressed by the value of the water contact angle was almost comparable to that reported for other PIs modified by spray-coating with a silylated substance (θ~160°) [45]. 

Contact angles exceeding 160° were reported for other types of polymer classes, namely polycarbonate nanofur [46,47] and fluorinated ethylene propylene [48]. Based on these studies, the examined PIs could be further developed by adapting the chemical surface modification procedure to adjust the PI morphology in such a manner that the superhydrophobicity improved to the level of other polymers discussed in the literature. In terms of light management the surface can also be textured as reported in another work on similar PI structures [28]. The observed surface features of PI samples were highly desirable to resolve the issues caused by impurity accumulation on the surface of the photovoltaic system, allowing more solar radiation to pass toward the junction of the device. The proposed procedure of the surface adaptation of polymer covers opens up fresh perspectives for solar cells that maintain their conversion efficiency at the desired level.

## 4. Conclusions

This paper provides important contributions regarding the replacement of the cover glass in solar cells. The solution consists of using polyimide layers that concomitantly diminish optical losses caused by TIR and surface soiling by means of their refraction, morphological, and wettability properties. Incorporating small cycloaliphatic CBDA moieties in combination with aromatic ones and chalcogen atoms in PI-1 and PI-4 leads to materials with a proper balance between refractivity (1.690–1.694) and transparency (>80%). The losses at the sample interface with ITO are diminished to about ~0.26% in regard to those achieved at a glass cover/ITO interface, which is around 0.97%. The polymers with the best transparency and refractivity are surface-treated by a new approach; it was shown that they gain a high level of hydrophobicity. AFM scans show that there is an evolution in the surface characteristics after each surface modification step, as supported by the changes in the root mean square roughness, surface area ratio, and surface-bearing index values. Surface wettability of the plasma-exposed and -sprayed surface of PI-1 is superhydrophobic (contact angle >150°); thus, optical losses caused by impurity accumulation are eliminated, while illuminance data reveal that there is no negative impact produced by the used surface treatment method. These results show that the proposed polymer materials exhibit adapted refractivity and surface-cleaning properties that improve the efficiency of solar cells by reducing optical losses caused by TIR and soiling. A potential disadvantage of this study is represented by the constant of the refractive index of the PI layer. In order to further minimize TIR-related losses, in a future investigation, it is recommended to prepare PI films with a gradient refractive index along the polymer thickness. This could be favorable for better matching the refraction features of PI with those of air and neighboring electrodes. 

## Figures and Tables

**Table 1:** The values of the vitrification temperature and coefficient of thermal expansion for the studied PIs.

Sample	Tg, K *	CTE, K^−1^
PI-1	615.200	16.33∙10^−5^
PI-2	480.140	20.64∙10^−5^
PI-3	477.250	20.76∙10^−5^
PI-4	513.730	19.37∙10^−5^

* Values taken from reference [27].

**Table 2:** Texture parameters calculated from the AFM height images for the pristine and surface-adapted PI-1 and PI-4 films.

Sample	Texture Parameters		
	Root Mean Square Roughness, Sq (nm)	Surface Area Ratio, Sdr (%)	Surface Bearing Index, Sbi
PI-1	2.3	0.081	0.032
PI-1(P)	10.4	0.326	0.084
PI-1(P+S)	64.2	3.493	0.413
PI-4	5.9	0.679	0.092
PI-4(P)	7.5	0.260	0.117
PI-4(P+S)	144.1	3.398	0.519

## Data Availability

Not applicable.

## References

[1] Sadasivuni K.K., Deshmukh K., Ahipa T.N., Muzaffar A., Ahamed M.B., Pasha S.K.K., Al-Maadeed M.A.-A. (2019). Flexible, biodegradable and recyclable solar cells: A review. J. Mater. Sci. Mater. Electron..

[2] Asim N., Sopian K., Ahmadi S., Saeedfar K., Alghoul M.A., Saadatian O., Zaidi S.H. (2012). A review on the role of materials science in solar cells. Renew. Sustain. Energy Rev..

[3] Li X., Li P., Wu Z., Luo D., Yu H.-Y., Lu Z.-H. (2021). Review and perspective of materials for flexible solar cells. Mater. Reports Energy.

[4] Feenstra J., Van Leest R.H., Smeenk N.J., Oomen G., Bongers E., Mulder P., Vlieg E., Schermer J.J. (2016). Flexible shielding layers for solar cells in space applications. J. Appl. Polym. Sci..

[5] Kim T., Kim J.-H., Kang T.E., Lee C., Kang H., Shin M., Wang C., Ma B., Jeong U., Kim T.-S. (2015). Flexible, highly efficient all-polymer solar cells. Nat. Commun..

[6] Yang W., Luo Z., Sun R., Guo J., Wang T., Wu Y., Wang W., Guo J., Wu Q., Shi M. (2020). Simultaneous enhanced efficiency and thermal stability in organic solar cells from a polymer acceptor additive. Nat. Commun..

[7] Hou G., García I., Rey-Stolle I. (2021). High-low refractive index stacks for broadband antireflection coatings for multijunction solar cells. Sol. Energy.

[8] Nayshevsky I., Xu Q.F., Barahman G., Lyons A.M. (2020). Fluoropolymer coatings for solar cover glass: Anti-soiling mechanisms in the presence of dew. Sol. Energy Mater. Sol. Cells.

[9] Sanchez-Friera P., Montiel D., Gil J.F., Montanez J.A., Alonso J. Daily Power Output Increase of Over 3% with the Use of Structured Glass in Monocrystalline Silicon PV Modules. Proceedings of the 2006 IEEE 4th World Conference on Photovoltaic Energy Conference.

[10] Sulaiman S.A., Singh A.K., Mokhtar M.M.M., Bou-Rabee M.A. (2014). Influence of Dirt Accumulation on Performance of PV Panels. Energy Procedia.

[11] Epure E.-L., Stoica I., Albu R.M., Hulubei C., Barzic A.I. (2021). New Strategy for Inducing Surface Anisotropy in Polyimide Films for Nematics Orientation in Display Applications. Nanomaterials.

[12] Sava I., Stoica I., Topala I., Mihaila I., Barzic A.I. (2022). Photodesign and fabrication of surface relief gratings on films of polyimide-based supramolecular systems obtained using host-guest strategy. Polymer.

[13] Yusoff A.R.M.M., Syahrul M.N., Henkel K. (2007). Film adhesion in amorphous silicon solar cells. Bull. Mater. Sci..

[14] Aydin E., Sankir N.D. (2017). Photovoltaic performance and impedance spectroscopy analysis of CuInS2 thin film solar cells deposited on polyimide foil via spray pyrolysis. Int. J. Electrochem. Sci..

[15] Dayneko S., Tameev A., Tedoradze M., Martynov I., Artemyev M., Nabiev I., Chistyakov A. (2013). Hybrid heterostructures based on aromatic polyimide and semiconductor CdSe quantum dots for photovoltaic applications. Appl. Phys. Lett..

[16] Niu H., Wang C., Bai X., Huang Y. (2004). New perylene polyimides containing p-n diblocks for sensitization in TiO_2_ solar cells. Polym. Adv. Technol..

[17] Hulubei C., Albu R.M., Lisa G., Nicolescu A., Hamciuc E., Hamciuc C., Barzic A.I. (2019). Antagonistic effects in structural design of sulfur-based polyimides as shielding layers for solar cells. Sol. Energy Mater. Sol. Cells.

[18] Yang C.P., Chen Y.P., Woo E.M., Li S.H. (2006). Light-color soluble polyimides based on α,α′-bis[4-(4- amino-2-trifluoromethylphenoxy)phenyl]-1,3-diisopropylbenzene and aromatic dianhydrides. Polym. J..

[19] Liu Y., Wang Y., Wu D. (2022). Synthetic strategies for highly transparent and colorless polyimide film. J. Appl. Polym. Sci..

[20] Mathews A.S., Kim I., Ha C. (2007). Synthesis, characterization, and properties of fully aliphatic polyimides and their derivatives for microelectronics and optoelectronics applications. Macromol. Res..

[21] Hu S., Shabani F., Liu B., Zhang L., Guo M., Lu G., Zhou Z., Wang J., Huang J.C., Min Y. (2022). High-Performance Deep Red Colloidal Quantum Well Light-Emitting Diodes Enabled by the Understanding of Charge Dynamics. ACS Nano.

[22] Choi W.-S., Park H.J., Park S.-H., Jeong T. (2014). Flexible InGaN LEDs on a Polyimide Substrate Fabricated Using a Simple Direct-Transfer Method. IEEE Photonics Technol. Lett..

[23] Liu Q., Feng Y., Tian H., He X., Hu A., Guo X. (2020). Fabrication of flexible AlGaInP LED. J. Semicond..

[24] Luo D., Chen Q., Liu B., Qiu Y. (2019). Emergence of Flexible White Organic Light-Emitting Diodes. Polymers.

[25] Barzic A.I. (2022). Novel aspects derived from the influence of dispersion properties of poly(4-vinylpyridine)/aluminum nitride nanocomposite encapsulants on light-extraction efficiency of light emitting diodes. Polym. Adv. Technol..

[26] Barzic A.I., Albu R.M., Hulubei C. (2021). Polyimides Containing Chalcogen Atoms in Solution Phase: Viscoelasticity and Interferometry Analyses. Rev. Roum. Chim..

[27] Barzic A.I., Albu R.M., Stoica I., Varganici C.D., Hulubei C. (2022). Polyimides containing cycloaliphatic units and chalcogen atoms as alternative shielding coatings for solar cells. Polym. Bull..

[28] Stoica I., Albu R.M., Hulubei C., Astanei D.G., Burlica R., Mersal G.A.M., Seaf Elnasr T.A., Barzic A.I., Elnaggar A.Y. (2022). A New Texturing Approach of a Polyimide Shielding Cover for Enhanced Light Propagation in Photovoltaic Devices. Nanomaterials.

[29] Green C.P., Lioe H., Cleveland J.P., Proksch R., Mulvaney P., Sader J.E. (2004). Normal and torsional spring constants of atomic force microscope cantilevers. Rev. Sci. Instrum..

[30] ISO *ISO 25178-2:2012*; Geometrical Product Specifications (GPS)—Surface texture: Areal—Part 2: Terms, Definitions and Surface Texture Parameters. https://www.iso.org/standard/42785.html.

[31] Lee S.J., Choi M.Y., Kwac L.K., Kim H.G., Chang J.-H. (2022). Comparison of Properties of Colorless and Transparent Polyimide Nanocomposites Containing Chemically Modified Nanofillers: Functionalized-Graphene and Organoclay. Polymers.

[32] Chang J.-H. (2020). Equibiaxially stretchable colorless and transparent polyimides for flexible display substrates. Rev. Adv. Mater. Sci..

[33] Tauc J., Menth A. (1972). States in the gap. J. Non. Cryst. Solids.

[34] Ayik C., Studenyak I., Kranjec M., Kurik M. (2014). Urbach Rule in Solid State Physics. Int. J. Opt. Appl..

[35] Jarzabek B., Schab-Balcerzak E., Chamenko T., Sek D., Cisowski J., Volozhin A. (2002). Optical properties of new aliphatic–aromatic co-polyimides. J. Non. Cryst. Solids.

[36] Rosenberg A., Lee S.H., Shirk J.S., Beadie G. (2018). Opto-thermal characteristics of amorphous polyimides for optical applications. Opt. Mater. Express.

[37] Jain V.K., Kulshreshtha A.P. (1981). Indium-Tin-Oxide transparent conducting coatings on silicon solar cells and their “figure of merit”. Sol. Energy Mater..

[38] Zhou Y., Shim J.W., Fuentes-Hernandez C., Sharma A., Knauer K.A., Giordano A.J., Marder S.R., Kippelen B. (2012). Direct correlation between work function of indium-tin-oxide electrodes and solar cell performance influenced by ultraviolet irradiation and air exposure. Phys. Chem. Chem. Phys..

[39] Bicerano J. (2002). Prediction of Polymers.

[40] Numata S., Oohara S., Imaizumi J., Kinjo N. (1985). Thermal Expansion Behavior of Various Aromatic Polyimides. Polym. J..

[41] Barzic A.I., Stoica I., Popovici D., Vlad S., Cozan V., Hulubei C. (2012). An insight on the effect of rubbing textile fiber on morphology of some semi-alicyclic polyimides for liquid crystal orientation. Polym. Bull..

[42] Stoica I., Barzic A.I., Hulubei C. (2017). Fabrication of nanochannels on polyimide films using dynamic plowing lithography. Appl. Surf. Sci..

[43] Popovici D., Barzic A.I., Stoica I., Butnaru M., Ioanid G.E., Vlad S., Hulubei C., Bruma M. (2012). Plasma modification of surface wettability and morphology for optimization of the interactions involved in blood constituents spreading on some novel copolyimide films. Plasma Chem. Plasma Process..

[44] Quan Y.-Y., Zhang L.-Z., Qi R.-H., Cai R.-R. (2016). Self-cleaning of Surfaces: The Role of Surface Wettability and Dust Types. Sci. Rep..

[45] Lo T.-Y., Huang Y.-C., Hsiao Y.-N., Chao C.-G., Whang W.-T. (2014). Preparation of superhydrophobic polyimide films modified with organosilicasol as effective anticorrosion coatings. Surf. Coat. Technol..

[46] Vüllers F., Gomard G., Preinfalk J.B., Klampaftis E., Worgull M., Richards B., Hölscher H., Kavalenka M.N. (2016). Bioinspired Superhydrophobic Highly Transmissive Films for Optical Applications. Small.

[47] Yan W., Huang Y., Wang L., Vüllers F., Kavalenka M.N., Hölscher H., Dottermusch S., Richards B.S., Klampaftis E. (2018). Photocurrent enhancement for ultrathin crystalline silicon solar cells via a bioinspired polymeric nanofur film with high forward scattering. Sol. Energy Mater. Sol. Cells.

[48] Roslizar A., Dottermusch S., Vüllers F., Kavalenka M.N., Guttmann M., Schneider M., Paetzold U.W., Hölscher H., Richards B.S., Klampaftis E. (2019). Self-cleaning performance of superhydrophobic hot-embossed fluoropolymer films for photovoltaic modules. Sol. Energy Mater. Sol. Cells.

